# VEXAS Mimicking Antiphospholipid Syndrome: Diagnostic Challenges of False-Positive Antiphospholipid Antibodies

**DOI:** 10.3390/ijms27146194

**Published:** 2026-07-11

**Authors:** Sayeed Khan, Hadia Eiman, Sanober Nusrat

**Affiliations:** 1 Department of Molecular and Cellular Physiology, Albany Medical College, Albany, NY 12208, USA; khans16@amc.edu; 2 Department of Medicine, Holy Family Hospital, Rawalpindi 46300, Punjab, Pakistan; hadiaeiman17@gmail.com; 3 Hematology-Oncology Section, Department of Internal Medicine, College of Medicine, University of Oklahoma Health Campus, Oklahoma City, OK 73104, USA

Somatic *UBA1*-mutated VEXAS syndrome is now recognized as a multisystem disorder marked by diffuse systemic inflammation, refractory cytopenia with or without bone marrow dysplasia, and thrombotic events affecting both venous and arterial systems [1]. Recurrent thrombosis often prompts evaluation for acquired thrombophilia, including antiphospholipid syndrome (APS). However, interpretation of antiphospholipid antibody testing may become challenging in the setting of profound inflammation, endothelial injury and concurrent anticoagulation—features inherent to VEXAS.

We report a 68-year-old man with transfusion-dependent macrocytic anemia, inflammatory arthritis, nodular skin lesions, and non-specific mediastinal lymphadenopathy who developed multiple superficial and deep venous thrombotic events. A lupus anticoagulant was persistently detected ≥12 weeks apart during a period of severe systemic inflammation (peak C-reactive protein 170 mg per liter) and ongoing anticoagulation, prompting an APS diagnosis and consideration for indefinite anticoagulation. The patient had been on rivaroxaban previously and has been on apixaban for the past several years. Baseline PTT on two occasions prior to APS evaluation measured 25.5–31.2 s (within normal limits); aPTT results proximate to the original APS diagnosis, made several years prior, were unavailable for review. Anti-cardiolipin and anti-ß2-glycoprotein I antibodies remained persistently negative on serial testing. Bone marrow evaluation for transfusion-dependent anemia revealed hypercellular trilineage hematopoiesis with vacuolated myeloid precursors and no clear evidence of dysplasia. Given a high index of clinical suspicion, genetic analysis was sent for *UBA1* p.Met41Thr pathogenic variant which confirmed VEXAS. Following Ruxolitinib therapy, inflammation improved substantially, allowing discontinuation of steroids, though transfusion dependence persisted. Momelotinib has now been initiated to evaluate whether a superior hemoglobin response can be achieved. Azacitidine remains a consideration for the next line of therapy.

This case raises several important diagnostic considerations. First, thrombosis in VEXAS is common, with venous events reported in up to 35–56% of patients and arterial events in up to 1–25% patients [2,3]. Given this baseline predisposition to thrombosis, we question whether an isolated lupus anticoagulant detected during intense systemic inflammation represents true APS or an epiphenomenon of the underlying autoinflammatory process. The prevalence of antiphospholipid antibodies in VEXAS syndrome has varied across studies: anticardiolipin antibodies and anti–β2-glycoprotein I antibodies were identified in 36% and 12% of tested patients, respectively. Another series reported persistently elevated lupus anticoagulant levels in 44% of patients [4,5]. Nevertheless, these estimates are limited by small cohorts, inconsistent testing methodologies, and the absence of confirmatory testing at ≥12 weeks as required by APS classification criteria. Moreover, lupus anticoagulant is particularly susceptible to false-positive results in the presence of marked systemic inflammation, direct oral anticoagulant exposure, or intercurrent infection, complicating interpretation of these findings [6,7]. Key distinguishing features between VEXAS syndrome and APS across clinical, laboratory, and diagnostic domains are summarized in Table 1.

Second, APS classification per the 2023 ACR/EULAR criteria requires both clinical criteria and persistent antibody positivity [8,9]. Our patient’s repeatedly negative anti-cardiolipin and anti-ß2-glycoprotein I antibodies argue against triple-positive APS. Accordingly, a formal APS diagnosis was not confirmed in this case; the persistently positive lupus anticoagulant is attributed to the inflammatory milieu of VEXAS syndrome rather than true antiphospholipid-mediated thrombophilia. This distinction carries therapeutic implications: while warfarin is strongly recommended for triple-positive patients and those with arterial events, the 2023 EULAR recommendations do not endorse direct oral anticoagulants for APS given prospective trial data demonstrating inferiority to warfarin. Anticoagulation decisions in VEXAS-associated thrombosis with equivocal aPL serology therefore remain individualized and poorly defined by existing guidelines.

Finally, whether VEXAS syndrome patients with thrombosis but equivocal antiphospholipid serology should receive APS-directed anticoagulation versus management based on inflammatory pathobiology of VEXAS syndrome itself remains uncertain. While anticoagulation is generally considered indefinite or long-term in patients with antiphospholipid syndrome, the optimal duration of anticoagulation in VEXAS syndrome remains undefined. Given the central role of systemic inflammation in VEXAS-associated thrombosis, discontinuation of anticoagulation may be considered on a case-by-case basis after sustained control of inflammatory activity and careful reassessment of thrombotic risk. We present this case to highlight the diagnostic complexity at the intersection of autoinflammatory bone marrow disorders and secondary thrombophilia, and to raise the question of how established APS criteria should be appropriately applied in this unique patient population.

## Figures and Tables

**Table 1:** Differential diagnosis: VEXAS syndrome vs. antiphospholipid syndrome.

Feature	VEXAS Syndrome	Antiphospholipid Syndrome (APS)
**EPIDEMIOLOGY and PATHOGENESIS**		
Etiology	Somatic *UBA1* mutation → ubiquitin pathway dysregulation → autoinflammation [1].	Autoimmune; antiphospholipid antibodies → endothelial activation → thrombosis.
Typical onset	Middle-aged to older adults; almost exclusively males [1].	Any age; female predominance in primary APS.
Hereditary	Somatic (acquired), not heritable [1].	Not directly heritable; familial clustering reported.
**CLINICAL FEATURES**		
Thrombosis	Venous 35–56%; arterial 1–25%; driven by systemic inflammation [2,3].	Venous and/or arterial thrombosis; defining clinical criterion.
Hematologic	Transfusion-dependent macrocytic anemia, thrombocytopenia, cytopenias.	Thrombocytopenia (~30%); hemolytic anemia (Evans syndrome) possible.
Skin	Nodular lesions, livedo racemosa, sweet syndrome-like eruptions.	Livedo reticularis; skin necrosis in severe APS.
Musculoskeletal	Inflammatory arthritis; relapsing polychondritis-like features.	Arthralgia/arthritis possible; less prominent.
Obstetric	Not a defining feature.	Recurrent pregnancy loss, pre-eclampsia, placental insufficiency (defining criterion).
Pulmonary	Pulmonary infiltrates, alveolar hemorrhage, pleural effusion.	Pulmonary embolism; pulmonary hypertension in chronic thromboembolic disease.
**LABORATORY FINDINGS**		
Lupus anticoagulant (LA)	Detected in up to 44%; frequently false-positive due to systemic inflammation or DOAC exposure [4,5].	Persistent positivity ≥12 weeks apart required for APS classification.
Anti-cardiolipin (aCL)	Positive in ~36%; not specific for APS in this context [4,5].	Medium-to-high titer positivity required (≥40 U or ≥99th percentile).
Anti-β2-glycoprotein I (aβ2GPI)	Positive in ~12%; generally low titer [4,5].	Positivity ≥99th percentile required for classification.
Inflammatory markers	Markedly elevated CRP, ESR, ferritin; characteristic of active disease.	May be mildly elevated; not a defining feature.
aPTT/coagulation	Baseline PTT typically normal; may be prolonged by LA effect or anticoagulant therapy.	Prolonged aPTT that does not correct on mixing study (LA effect).
**DIAGNOSTIC CRITERIA**		
Confirmatory test	Somatic *UBA1* pathogenic mutation on targeted sequencing [1].	Persistent aPL positivity (≥1 of 3 antibodies) ≥12 weeks apart plus clinical criterion (2023 ACR/EULAR criteria) [8].
Bone marrow biopsy	Vacuolated myeloid and erythroid precursors; dysplasia may be absent [1].	Not required; may show changes in secondary APS (e.g., SLE-associated).
Triple antibody positivity	Rare; high false-positive risk in setting of active inflammation and anticoagulation [4,5].	LA + aCL + aβ2GPI triple positivity associated with highest thrombotic risk.
**MANAGEMENT**		
Anticoagulation	Indicated for thrombotic events; optimal duration uncertain; may be individualized with inflammation control.	Long-term/indefinite anticoagulation; warfarin preferred for triple-positive or arterial events; DOACs not recommended per 2023 EULAR guidelines [8].
Disease-modifying therapy	Azacitidine, ruxolitinib/JAK inhibitors; corticosteroids for acute inflammation; allogeneic SCT in select cases.	Hydroxychloroquine (in secondary APS/SLE); immunosuppression for refractory cases.
Monitoring	Serial CBC, inflammatory markers, bone marrow evaluation; hematology-driven.	Serial aPL antibody titers; anticoagulation monitoring (INR for warfarin).

Abbreviations: aPL—antiphospholipid antibody; aCL—anti-cardiolipin; aβ2GPI—anti-β2-glycoprotein I; APS—antiphospholipid syndrome; CBC—complete blood count; CRP—C-reactive protein; DOAC—direct oral anticoagulant; ESR—erythrocyte sedimentation rate; LA—lupus anticoagulant; SCT—stem cell transplant; SLE—systemic lupus erythematosus; *UBA1*—ubiquitin-like modifier activating enzyme 1; VEXAS—vacuoles, E1 enzyme, X-linked, autoinflammatory, somatic.

## Data Availability

No new data were created or analyzed in this study. Data sharing is not applicable to this article.
